# Long-term safety and efficacy of pegvaliase for the treatment of phenylketonuria in adults: combined phase 2 outcomes through PAL-003 extension study

**DOI:** 10.1186/s13023-018-0858-7

**Published:** 2018-07-04

**Authors:** Nicola Longo, Roberto Zori, Melissa P. Wasserstein, Jerry Vockley, Barbara K. Burton, Celeste Decker, Mingjin Li, Kelly Lau, Joy Jiang, Kevin Larimore, Janet A. Thomas

**Affiliations:** 1 Department of Pediatrics, Division of Medical Genetics, University of Utah, 295 Chipeta Way, Salt Lake City, UT 84108 USA; 2 Division of Genetics and Metabolism, University of Florida, PO Box 100296 UFHSC, Gainesville, FL 32610 USA; 3 Department of Pediatrics, The Children’s Hospital at Montefiore, 3415 Bainbridge Ave, Bronx, NY 10467 USA; 4 Department of Pediatrics, Division of Medical Genetics, University of Pittsburgh and Children’s Hospital of Pittsburgh, 4401 Penn Avenue, Pittsburgh, PA 15224 USA; 5 Department of Pediatrics, Division of Genetics, Birth Defects & Metabolism, Ann & Robert H. Lurie Children’s Hospital of Chicago, 225 E. Chicago Ave, Chicago, IL 60611 USA; 6 BioMarin Pharmaceutical Inc, 105 Digital Drive, Novato, CA 94949 USA; 7 Department of Pediatrics, Section of Clinical Genetics and Metabolism, University of Colorado Hospital, 12605 E. 16th St, Aurora, CO 80045 USA

**Keywords:** Phenylketonuria, PKU, Recombinant *Anabaena variabilis* PEGylated phenylalanine ammonia lyase, Pegvaliase

## Abstract

**Background:**

Deficiency of phenylalanine hydroxylase causes phenylketonuria (PKU) with elevated phenylalanine (Phe) levels and associated neuropsychiatric and neurocognitive symptoms. Pegvaliase (PEGylated phenylalanine ammonia lyase) is an investigational agent to lower plasma Phe in adults with PKU. This study aimed to characterize the long-term efficacy, safety, and immunogenicity of pegvaliase in adults with PKU.

**Methods:**

PAL-003 is an ongoing, open-label, long-term extension study of the pegvaliase dose-finding parent phase 2 studies. Participants continued the dose of pegvaliase from one of three parent studies, with dose adjustments to achieve a plasma Phe concentration between 60 and 600 μmol/L.

**Results:**

Mean (standard deviation [SD]) plasma Phe at treatment-naïve baseline for 80 participants in the parent studies was 1302.4 (351.5) μmol/L. In the 68 participants who entered the extension study, plasma Phe decreased 58.9 (39)% from baseline, to 541.6 (515.5) μmol/L at Week 48 of treatment. Plasma Phe concentrations ≤120 μmol/L, ≤360 μmol/L, and ≤ 600 μmol/L were achieved by 78.7, 80.0, and 82.5% of participants, respectively. Mean (SD) protein intake at baseline was 69.4 (40.4) g/day (similar to the recommended intake for the unaffected population) and remained stable throughout the study. All participants experienced adverse events (AEs), which were limited to mild or moderate severity in most (88.8%); the most common AEs were injection-site reaction (72.5%), injection-site erythema (67.5%), headache (67.5%), and arthralgia (65.0%). The AE rate decreased from 58.3 events per person-year in the parent studies to 18.6 events per person-year in the extension study.

**Conclusions:**

Pegvaliase treatment in adults with PKU produced meaningful and persistent reductions in mean plasma Phe concentration with a manageable safety profile for most subjects that continued with long-term treatment.

**Trial registration:**

ClinicalTrials.gov, NCT00924703. Registered June 18, 2009, https://clinicaltrials.gov/ct2/show/NCT00924703

**Electronic supplementary material:**

The online version of this article (10.1186/s13023-018-0858-7) contains supplementary material, which is available to authorized users.

## Background

Phenylketonuria (PKU; OMIM 261600) is an autosomal recessive disorder with an incidence in the United States of approximately 1 in 16,500 newborns [1]. PKU is caused by a deficiency of phenylalanine hydroxylase, the enzyme that converts phenylalanine (Phe) to tyrosine, resulting in an accumulation of Phe in the blood and brain [2].

In some patients, chronically elevated Phe levels can lead to a range of developmental, neurocognitive, and neuropsychiatric comorbidities [3]. Despite advances in treatment, 20–65% of adults with PKU fail to achieve metabolic control. This stems from a number of factors, including issues with adherence to treatment, access to care, and the limited efficacy of available therapeutic interventions [4–8].

Guidelines from the American College of Medical Genetics and Genomics (ACMG) recommend lifelong treatment to maintain plasma Phe concentration between 120 μmol/L and 360 μmol/L to minimize the adverse impact of elevated Phe on the brain [2]. Treatment goals related to plasma Phe are individualized based on the impact of high Phe concentrations on patients [9]. Individuals with PKU are counseled to lower plasma Phe with a diet severely restricted in proteins and supplemented with low-Phe or Phe-free, amino acid–modified medical foods or special low-protein foods, with or without sapropterin dihydrochloride (KUVAN®, BioMarin Pharmaceutical Inc., Novato, CA), which is indicated for the treatment of PKU in combination with diet [2, 8]. Pegvaliase (Palynziq™, BioMarin Pharmaceutical Inc.) is a Phe-metabolizing enzyme recently approved in the US to reduce plasma Phe concentrations in adults with PKU who have plasma Phe concentrations > 600 μmol/L [10].

Pegvaliase is a recombinant *Anabaena variabilis* phenylalanine ammonia lyase (PAL) enzyme conjugated to polyethylene glycol (PEG) to optimize pharmacodynamic stability and reduce immune response to PAL [11]. PAL converts Phe to ammonia and trans-cinnamic acid, which are readily metabolized by the liver and excreted in the urine [10–12].

The phase 1 study PAL-001, a dose-escalation study of pegvaliase in adults with PKU, showed that a single dose of 0.1 mg/kg reduced plasma Phe levels from baseline and maintained the reduction for up to 1 week [11]. The first phase 2 study, PAL-002, a dose-finding study of pegvaliase, demonstrated that once-weekly dosing at 0.001 mg/kg to 0.1 mg/kg for 16 weeks was generally well tolerated, but did not result in significant plasma Phe reductions. In the subsequent phase 2 study PAL-004, pegvaliase at 0.06 mg/kg to 0.4 mg/kg for 5 days/week caused immediate and substantial plasma Phe reductions. However, many participants had to reduce or interrupt pegvaliase due to hypersensitivity events, and plasma Phe reduction was not sustained at the end of 13 weeks of treatment [13]. In the third phase 2 study, 165–205, pegvaliase was started at a low dose (2.5 mg/week) that was gradually increased (to a maximum of 375 mg/week) to minimize hypersensitivity events. This pegvaliase dosing regimen with an induction, titration, and maintenance period led to substantial reductions in mean plasma Phe concentration during the 24-week study [14].

Here we report on the longest duration of pegvaliase treatment to date. Long-term efficacy, safety, and immunogenicity of pegvaliase was assessed in participants who initiated treatment in PAL-002 (NCT00925054) [13], PAL-004 (NCT01212744) [13], or 165–205 (NCT01560286) [14] and then continued on in the PAL-003 extension study reported here.

In addition to the studies described above, two phase 3 clinical trials of pegvaliase, which included a double-blind, randomized withdrawal trial [15] and an open-label, long-term extension study [16], were recently published. These phase 3 studies evaluated the efficacy and safety of pegvaliase dosed at 5 to 60 mg/day.

## Methods

### Study design

PAL-003 is an open-label, multicenter, long-term phase 2 extension study of pegvaliase treatment in adults with PKU who completed a prior dose-finding parent phase 2 study. The primary objective of this study was to evaluate the long-term effect of pegvaliase on plasma Phe concentration; secondary objectives were to evaluate safety and immune response; and an exploratory objective was to assess the relationship between dietary protein intake and changes in plasma Phe. PAL-003 began on January 5, 2010, and we report here data acquired through October 27, 2016.

### Study participants

Individuals with PKU who completed a parent study with pegvaliase and were willing and able to maintain stable protein intake were eligible to enroll in the extension study. Sexually active participants were required to use two acceptable methods of contraception.

Patients were excluded if they were taking, other than pegvaliase, any medication to treat PKU or any injectable drugs containing PEG (e.g., medroxyprogesterone). Patients currently pregnant or planning to become pregnant (self or partner) or breastfeed during the study period were excluded. Patients who had a prior systemic hypersensitivity event (e.g., generalized hives, hypotension, anaphylaxis, or angioedema) to a PEG-containing product were excluded, with the exception that patients with a prior reaction to pegvaliase could be eligible for participation at the discretion of the principal investigator in consultation with the Sponsor’s medical monitor.

### Study drug administration

Participants who enrolled in the extension study either continued their pegvaliase dosing regimen from their parent study or started at a higher dose, as determined by the primary investigator and medical monitor. The dose level and/or frequency were adjusted for each participant to achieve a plasma Phe concentration between 60 and 600 μmol/L or in response to an adverse event (AE).

Pegvaliase doses were similar to those previously tested in the parent studies [13, 14], between 2.5 mg/week and 375 mg/week or between 0.001 mg/kg/week and 5 mg/kg/week, administered up to 7 days/week; weight-based dosing allowed participants > 75 kg to receive a dose higher than 375 mg/week, but a protocol change in October 2014 clarified an absolute maximum dose of 375 mg/week.

The first dose of pegvaliase in the extension study was administered in the clinic. Injection sites were rotated between doses in common areas for subcutaneous injection (e.g., upper arm, thigh, or abdomen).

### Dietary intake

Adherence to a Phe-restricted diet was not required, but participants were instructed to maintain a total protein intake consistent with their dietary intake at time of entry into the extension study. This was so that changes in endpoints would be attributable to the study drug rather than to changes in protein intake. When necessary in patients with low Phe levels, protein intake was increased under the supervision of the investigator. Average daily dietary protein and Phe intake was calculated using 3-day diet records before study visits.

### Hypersensitivity adverse events

Participants were trained to recognize and respond to hypersensitivity AEs (HAEs) and were given epinephrine injectors for use in the event of an acute systemic hypersensitivity event, including potential anaphylaxis events. Investigators used clinical judgment to decide if a participant should receive premedication with a histamine receptor-1 antagonist, histamine receptor-2 antagonist, and/or antipyretic prior to subsequent pegvaliase dosing. In the event of an acute systemic hypersensitivity event, including potential anaphylaxis events, the dose of pegvaliase could be maintained, reduced, or interrupted at the discretion of the investigator. Starting in October 2014, in the event of acute systemic hypersensitivity that met Brown’s severe criteria (i.e., hypoxia, hypotension, or neurologic compromise) [17], pegvaliase was withdrawn.

### Assessments

Safety and plasma Phe concentration were assessed every 4 weeks and immune response every 12 weeks. A central laboratory was used to analyze plasma Phe concentrations.

Safety was assessed by vital signs, physical examination, AEs, and clinical laboratory tests (chemistry, hematology, and urinalysis). The incidence, exposure-adjusted event rate, and severity grade (per National Cancer Institute Common Terminology Criteria for Adverse Events [CTCAE], version 4.03 [18]: mild, moderate, severe, life-threatening, or death) of AEs were reported.

All AEs were coded according to Medical Dictionary for Regulatory Activities (MedDRA; version 18.0 [19]) preferred terms. AEs of special interest for purposes of additional safety monitoring included injection-site reactions, generalized skin reactions with duration ≥14 days, arthralgia, HAEs, and acute systemic hypersensitivity events, which were identified using predefined search strategies. All potential acute systemic hypersensitivity events and AEs where epinephrine was administered were reviewed by an allergist/immunologist independent of the clinical site and Sponsor to identify acute systemic hypersensitivity events consistent with clinical anaphylaxis criteria as defined by National Institute of Allergy and Infectious Diseases/Food Allergy and Anaphylaxis Network (NIAID/FAAN) and Brown’s severe criteria [17]. In addition to the Sponsor, an independent Data Monitoring Committee monitored the safety of participants, acting in an advisory capacity to the Sponsor.

Immunology testing was performed using validated assays to measure immunoglobulin G (IgG) and immunoglobulin M (IgM) responses to PAL, PEG, and neutralizing antibodies (NAbs). Serum samples for routine immunology testing were collected prior to dosing. In the event of an acute systemic hypersensitivity event, participants may have had an extra clinic visit scheduled, at the discretion of the investigator, at which samples were collected for anti-PAL and anti-pegvaliase immunoglobulin E (IgE) measurement.

### Statistical analysis

Descriptive summaries of continuous variables (i.e., number of participants [n], mean, standard deviation [SD], median, and range) and of categorical variables (i.e., n and percentage) were analyzed by parent studies (all parent study data pooled together), the extension study, and overall (cumulative phase 2 treatment) to provide a robust population for evaluating trends in efficacy and safety outcomes with long-term treatment. Population mean data were summarized over time at intervals relative to baseline. The baseline value was defined as the last measurement before the first dose of pegvaliase (i.e., while patients were treatment naïve) in the parent study, unless indicated otherwise. The pegvaliase weekly dose was defined as the sum of the doses in the 7 days prior to each assessment. Daily dose categories were calculated using total drug amount (mg) divided by entire duration (days) of study to allow comparison of dose between studies.

The efficacy population included all participants who received at least one dose of pegvaliase and had at least one post-treatment plasma Phe concentration measurement. Plasma Phe measurements up to 14 days after a pegvaliase dose were included. Data presented as cumulative weeks of treatment include parent studies and the extension study.

The safety population included all participants who received at least one dose of pegvaliase [13, 14]. For AEs occurring more than once in a participant during the study, the AE of maximum severity was used in summaries. The duration of the parent studies was much shorter (up to 24 weeks of pegvaliase dosing) than the extension study (up to 102 months of pegvaliase dosing) [13, 14]; therefore, event rates per person-years, adjusting for duration of exposure, are included for AE comparisons between parent and extension studies. Event rate comparisons between early (< 24 weeks) and late (> 24 weeks) treatment were made between different study populations (i.e., participants who discontinued the study during the first 24 weeks were not included in the late-treatment safety analysis).

The incidence rates of antibody positivity of PAL IgM, PAL IgG, PEG IgM, PEG IgG, and NAbs were summarized by treatment week. Antibody positivity, presented as percentage of participants, was calculated as the number of participants testing positive for an antibody divided by the total number of participants with data available at each timepoint. Plots were generated to explore the potential relationship between the incidence of antidrug antibodies and HAE frequency.

## Results

### Participant disposition

Sixteen investigators participated at 14 study centers in the United States. Eighty participants enrolled in one of the three parent studies, and 68 of these participants enrolled in the extension study. As of the cutoff date for data analysis, 37 participants remained in the extension study and 20 participants had discontinued the extension study (*n* = 10 due to withdrawal by participant decision, *n* = 5 due to physician decision, *n* = 3 lost to follow-up, and *n* = 2 due to an AE). Eleven participants completed the extension study and transferred into the phase 3 study PRISM-2.

### Participant baseline and demographic characteristics

There were no meaningful differences in demographics or baseline characteristics between participants enrolled in the parent studies and the subset who continued into the extension study (Table 1 and Additional file 1: Table S1). Baseline mean (SD) protein intake was 69.4 (40.4) g/day. Baseline mean (SD) plasma Phe concentration for phase 2 participants was 1302.4 (351.5) μmol/L; in participants who enrolled into the extension study, plasma Phe at entry to the extension study was 1022.4 (530.4) μmol/L.

**Table 1:** Participant demographic and baseline characteristics

	Phase 2 participants (*N* = 80)
Age at enrollment	
Mean (SD), years	28.3 (8.8)
Min, max, years	16, 56
< 18, n (%)	3 (3.8%)
≥ 18, n (%)	77 (96.3%)
Sex	
Female, *n* (%)	46 (57.5%)
Race	
White, *n* (%)	78 (97.5%)
Weight	*n* = 79
Mean (SD), kg	80.4 (24.5)
Min, max, kg	42.0, 178.0
Height	*n* = 74
Mean (SD), cm	167.2 (9.6)
Min, max, cm	149.8, 187.5
Body mass index	n = 74
Mean (SD), kg/m^2^	28.5 (7.7)
Min, max, kg/m^2^	17.2, 56.2
< 25 kg/m^2^, *n* (%)	28 (35.0%)
25 to < 30 kg/m^2^, *n* (%)	21 (26.3%)
≥ 30 kg/m^2^, *n* (%)	25 (31.3%)
Plasma Phe	
Mean (SD), μmol/L	1302.4 (351.5)
Min, max, μmol/L	249.0, 2214.0
Protein intake^a^	*n* = 36
Mean (SD), g/day	69.4 (40.0)
Min, max, g/day	10.7, 197.3
Phe intake^b^	n = 36
Mean (SD), mg/day	1975 (1583)
Min, max, mg/day	461, 8419

Baseline was defined as the last measurement before the first dose of pegvaliase (i.e., while patients were treatment naïve) in the parent study. All phase 2 data are included. Sample size is indicated if *N* < 80

*max* maximum, *min* minimum, *Phe* phenylalanine, *SD* standard deviation

^a^Protein intake includes medical food and natural protein dietary intake and was calculated as the daily average intake over 3 days prior to the assessment point

^b^Phe intake was calculated as the daily average intake over 3 days prior to the assessment point

### Pegvaliase exposure

The mean dose of pegvaliase increased from 5.3 (6.8) mg/day in the parent studies to 26.2 (17.9) mg/day in the extension study (Additional file 1: Table S2). Overall mean treatment duration in phase 2 studies was 167.0 weeks (approximately 3.4 years), providing a total of 256 total patient-years of exposure (Table 2). Approximately 66.3% of participants (53 of 80) received pegvaliase for at least 2 years and 38.8% (31 of 80) received treatment for at least 4 years.

**Table 2:** Pegvaliase exposure

	Phase 2 participants (*N* = 80)
Treatment duration, weeks	
Mean (SD)	167.0 (111.1)
Min, max	0.71, 370.4
Treatment duration, *n* (%)	
< 1 year	15 (18.8%)
≥ 1 to < 2 years	12 (15.0%)
≥ 2 to < 3 years	10 (12.5%)
≥ 3 to < 4 years	12 (15.0%)
≥ 4 years	31 (38.8%)

All data from phase 2 studies are included

*max* maximum, *min* minimum, *SD* standard deviation

### Efficacy

Mean (SD) plasma Phe decreased by 58.9% (39.0) from baseline to 541.6 (515.5) μmol/L at Week 48 of treatment and by 72.3% (37.5) to 372.0 (514.3) μmol/L at Week 120 (Additional file 1: Table S3).

**Fig. 1:**
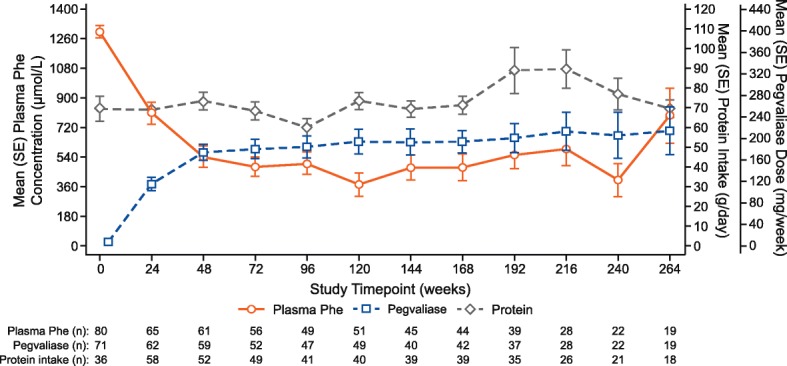
Plasma Phe concentration, pegvaliase dose, and protein intake over time. Protein intake includes medical food and natural protein intake and was calculated as the daily average of intake over 3 days prior to the assessment point. Data are presented as mean (SE). All phase 2 data are included. Sample size reflects participants with data available at study timepoint; study is ongoing. Abbreviations: *Phe* phenylalanine, *SE* standard error

A total of 78.7, 80.0, and 82.5% of participants achieved a plasma Phe concentration ≤ 120 μmol/L, ≤360 μmol/L, and ≤ 600 μmol/L, respectively, at any time during the study (Table 3). Protein intake remained relatively constant during the trial (Fig. 1).

**Table 3:** Participants with plasma Phe ≤120 μmol/L, ≤360 μmol/L, or ≤ 600 μmol/L

	*n*	Plasma Phe threshold, n (%)		
		≤120 μmol/L	≤360 μmol/L	≤600 μmol/L
Overall^a^	80	63 (78.7%)	64 (80.0%)	66 (82.5%)
Extension study				
Week 48	61	20 (32.8%)	28 (45.9%)	35 (57.4%)
Week 96	49	17 (34.7%)	22 (44.9%)	30 (61.2%)
Week 144	45	19 (42.4%)	25 (55.6%)	29 (64.4%)
Week 192	39	13 (33.3%)	17 (43.6%)	23 (58.9%)
Week 240	22	11 (50.0%)	13 (59.1%)	16 (72.7%)
Week 264	19	5 (26.3%)	6 (31.6%)	8 (42.1%)
Overall	68	57 (83.8%)	58 (85.2%)	59 (86.7%)

All data from phase 2 studies are included. Sample size reflects participants with data available at study timepoint; study is ongoing

*Phe* phenylalanine

^a^Achievement of thresholds at any time during phase 2 study

**Fig. 2:**
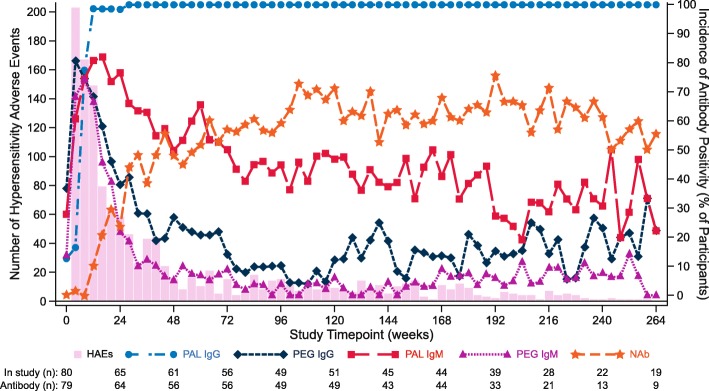
Frequency of hypersensitivity events and incidence of antibody positivity over time. Antibody positivity is calculated as the number of participants testing positive divided by the total number of participants at each study visit. All phase 2 data are included. Sample size reflects participants with data available at study timepoint; study is ongoing. Abbreviations: *IgG* immunoglobulin G, *IgM* immunoglobulin M, *PAL* phenylalanine ammonia lyase, *PEG* polyethylene glycol, *NAb* neutralizing antibody

Mean plasma Phe concentration decreased as the mean pegvaliase dose was increased over time (Fig. 2). Both mean plasma Phe concentration and the mean weekly dose of pegvaliase remained relatively stable from Week 48 to Week 264 (almost 5 years) of pegvaliase treatment. Mean daily protein intake remained relatively stable throughout the study.

### Safety

All participants reported at least one AE, with the most common AEs (by preferred term) being injection-site reaction (72.5% of participants), injection-site erythema (67.5%), headache (67.5%), and arthralgia (65.0%; Table 4). The majority of participants had AEs limited to mild (12.5%) or moderate (76.3%) severity; severe AEs were reported in 11.3% of participants. The overall AE event rate was 22.5 events per person-year; the event rate decreased during the extension study as compared to the parent studies (dropping from 58.3 to 18.6 events per person-year). There were no deaths (Table 5).

**Table 4:** AEs (incidence ≥25% all participants) reported as subject incidence (n, %), and event rate (events/person-years) and total number of events

Exposure, person-years	Parent study (*n* = 80)		Extension study (*n* = 68)		Overall phase 2 treatment (*n* = 80)^a^	
		25.1		230.0		256.0
	Incidence *n* (%)	Event rate (total number of events)	Incidence *n* (%)	Event rate (total number of events)	Incidence *n* (%)	Event rate (total number of events)
AEs	79 (98.8%)	58.3 (1463)	68 (100%)	18.6 (4284)	80 (100%)	22.5 (5747)
Headache	33 (41.3%)	2.8 (69)	43 (63.2%)	0.97 (223)	54 (67.5%)	1.1 (292)
Nasopharyngitis	15 (18.8%)	0.6 (15)	40 (58.8%)	0.43 (98)	47 (58.8%)	0.44 (113)
Rash	22 (27.5%)	1.3 (32)	35 (51.5%)	0.51 (118)	46 (57.5%)	0.59 (150)
Injection-site reaction	45 (56.3%)	9.1 (229)	35 (51.5%)	0.50 (115)	58 (72.5%)	1.3 (344)
Arthralgia	41 (51.3%)	5.6 (140)	34 (50.0%)	0.74 (171)	52 (65.0%)	1.2 (311)
Injection-site erythema	36 (45.0%)	3.6 (91)	34 (50.0%)	0.68 (157)	54 (67.5%)	0.97 (248)
Injection-site bruising	21 (26.3%)	1.2 (30)	33 (48.5%)	1.0 (240)	47 (58.8%)	1.1 (270)
Upper respiratory tract infection	17 (21.3%)	0.72 (18)	31 (45.6%)	0.64 (147)	39 (48.8%)	0.65 (165)
Oropharyngeal pain	11 (13.8%)	0.48 (12)	31 (45.6%)	0.28 (64)	37 (46.3%)	0.30 (76)
Urticaria	8 (10.0%)	0.44 (11)	30 (44.1%)	1.2 (281)	33 (41.3%)	1.1 (292)
Nausea	24 (30.0%)	1.3 (33)	30 (44.1%)	0.27 (62)	41 (51.3%)	0.37 (95)
Cough	15 (18.8%)	0.68 (17)	30 (44.1%)	0.20 (45)	39 (48.8%)	0.24 (62)
Back pain	5 (6.3%)	0.40 (10)	29 (42.6%)	0.30 (70)	32 (40.0%)	0.31 (80)
Pruritus	15 (18.8%)	0.92 (23)	27 (39.7%)	0.29 (66)	33 (41.3%)	0.35 (89)
Diarrhea	12 (15.0%)	0.68 (17)	25 (36.8%)	0.30 (70)	31 (38.8%)	0.34 (87)
Vomiting	9 (11.3%)	0.76 (19)	24 (35.3%)	0.22 (51)	28 (35.0%)	0.27 (70)
Contusion	5 (6.3%)	0.20 (5)	23 (33.8%)	0.24 (56)	26 (32.5%)	0.24 (61)
Pain in extremity	14 (17.5%)	0.76 (19)	23 (33.8%)	0.23 (52)	30 (37.5%)	0.28 (71)
Pyrexia	15 (18.8%)	0.76 (19)	23 (33.8%)	0.15 (35)	33 (41.3%)	0.21 (54)
Injection-site pruritus	9 (11.3%)	0.60 (15)	21 (30.9%)	0.32 (73)	25 (31.3%)	0.34 (88)
Myalgia	9 (11.3%)	0.44 (11)	21 (30.9%)	0.28 (64)	26 (32.5%)	0.29 (75)
Sinusitis	5 (6.3%)	0.20 (5)	21 (30.9%)	0.14 (32)	24 (30.0%)	0.15 (37)
Dizziness	20 (25.0%)	1.2 (30)	20 (29.4%)	0.22 (51)	36 (45.0%)	0.32 (81)
Abdominal pain	8 (10.0%)	0.44 (11)	18 (26.5%)	0.16 (36)	22 (27.5%)	0.18 (47)

Event rate was calculated as total number of events divided by person-years of exposure. Incidence rates counted participants who reported more than 1 adverse event within a preferred term only once

*AE* adverse event

^a^All phase 2 data are included

**Table 5:** AEs reported by subject incidence (n, %), event rate (events/person-years), and total number of events

Exposure, person-years	Parent study (*n* = 80)		Extension study (*n* = 68)		Overall phase 2 treatment (*n* = 80)^a^	
		25.1		230.0		256.0
	Incidence *n* (%)	Event rate (total number of events)	Incidence *n* (%)	Event rate (total number of events)	Incidence *n* (%)	Event rate (total number of events)
AEs	79 (98.8%)	58.3 (1463)	68 (100%)	18.6 (4284)	80 (100%)	22.5 (5747)
AEs causing early pegvaliase discontinuation	5 (6.3%)	0.20 (5)	4 (5.9%)	0.05 (12)	9 (11.3%)	0.07 (17)
SAEs	4 (5.0%)	0.16 (4)	11 (16.2%)	0.07 (16)	15 (18.8%)	0.08 (20)
SAEs causing early pegvaliase discontinuation	1 (1.3%)	0.04 (1)	2 (2.9%)	0.01 (3)	3 (3.8%)	0.02 (4)
Death	0	0	0	0	0	0
AEs of special interest						
Hypersensitivity events	69 (86.3%)	14.9 (373)	62 (91.2%)	4.3 (997)	75 (93.8%)	5.4 (1370)
Generalized skin reaction (≥14 days)	9 (11.3%)	0.44 (11)	37 (54.4%)	0.40 (93)	39 (48.8%)	0.41 (104)
Injection-site reaction	68 (85.0%)	19.1 (479)	59 (86.8%)	3.8 (875)	76 (95.0%)	5.3 (1354)
Injection-site skin reaction (≥14 days)	13 (16.3%)	0.88 (22)	25 (36.8%)	0.27 (62)	36 (45.0%)	0.33 (84)
Arthralgia	41 (51.3%)	5.6 (140)	34 (50.0%)	0.74 (171)	52 (65.0%)	1.2 (311)
Acute systemic hypersensitivity event of anaphylaxis	3 (3.8%)	15.9 (4)	1 (1.5%)	0.01 (2)	3 (3.8%)	0.02 (6)
Acute systemic hypersensitivity event per Brown’s severe criteria	1 (1.3%)	0.04 (1)	1 (1.5%)	0.01 (2)	2 (2.5%)	0.01 (3)

Event rate was calculated as total number of events divided by person-years of exposure

*AE* adverse event, *SAE* serious adverse event

^a^All phase 2 data are included

#### Extension study

Twenty-five participants (36.8%) reported AEs that led to dose interruption or reduction; the most common AEs by preferred term were urticaria (*n* = 5 [7.4% of participants]); arthralgia, generalized rash, hypersensitivity, and nausea (*n* = 3 [4.4% of participants] each); and pruritus, injection-site rash, injection-site reaction, presyncope, diarrhea, viral gastroenteritis, gastrointestinal viral infection, nasopharyngitis, and viral infection (*n* = 2 [2.9% of participants] each). The percentage of participants experiencing AEs was similar across dose groups (93.2% with doses < 20 mg/day; 83.6% with doses ≥20 to < 40; 86.3% with doses ≥40 to < 60; 84.4% with doses ≥60 mg/day).

Four participants (5.9%) had AEs leading to early discontinuation of pegvaliase treatment; two of these participants also discontinued study participation due to AEs. One participant with two previous acute systemic hypersensitivity events of anaphlyaxis during parent study treatment had two additional events of anaphylaxis in the extension study and discontinued treatment due to AEs after the fourth event (acute systemic hypersensitivity events further described below); one participant discontinued treatment due to AEs of severe arthralgia and peripheral neuropathy; one participant withdrew consent and discontinued treatment after experiencing mild pruritus; and one participant with four cases of presyncope discontinued treatment after the fourth event. All AEs resolved.

Eleven (16.2%) participants had a total of 16 serious AEs (SAEs) in the extension study, with two participants (2.9%) reporting SAEs by preferred term of asthma; other SAEs were reported by one participant each: anaphylactic reaction (associated with a confirmed acute systemic hypersensitivity event described below), appendicitis, infectious diarrhea, hypotension, lymphadenopathy, staphylococcal infection, and suicide attempt; hypersensitivity and asthma in one participant; injection-related reaction, neutropenia, and bone marrow failure in one participant; and asthma, arthralgia, and peripheral neuropathy in one participant. All SAEs resolved.

The most common AEs by preferred term in the extension study were headache (63.2%), nasopharyngitis (58.8%), rash (51.5%), injection-site reaction (51.5%), arthralgia (50.0%), and injection-site erythema (50.0%). The most common AEs by event rate were urticaria (1.2 events/person-year) and injection-site bruising (1.0 events/person-year).

#### Acute systemic hypersensitivity events

One participant in the extension study experienced two acute systemic hypersensitivity events of anaphylaxis consistent with clinical NIAID/FAAN criteria as confirmed by an independent allergist/immunologist, both of which also met Brown’s severe criteria. The coded AE preferred terms associated with these two events were hypersensitivity (*n* = 1) and anaphylactic reaction (n = 1). Both cases resolved without sequelae, and the participant was not admitted to the hospital. The participant resumed pegvaliase dosing 3 days after the first acute systemic hypersensitivity event in the extension study and continued pegvaliase for 150 days, at which point the second event occurred and the participant discontinued pegvaliase. Drug-specific IgE was not detected at or near the time of the acute systemic hypersensitivity events.

#### AEs of interest

At least one HAE was reported by 62 of 68 participants (91.2%) in the extension study. HAEs with the highest incidence in the extension study included rash (51.5%), arthralgia (50.0%), urticaria (44.1%), pruritus (39.7%), and pyrexia (33.8% [Tables 4 and 5]).

Severe HAEs occurring in the extension study included arthralgia (*n* = 2), asthma (n = 1), and anaphylactic reaction (n = 1, associated with a confirmed acute systemic hypersensitivity event described above). The frequency of HAEs decreased in the extension study compared to the parent studies, with an exposure-adjusted HAE rate of 14.9 events/person-year in the parent studies and 4.3 events/person-year in the extension study. The frequency of injection-site reactions, generalized skin reaction lasting ≥14 days, injection-site skin reaction lasting ≥14 days, and arthralgia also decreased in the extension study compared to the parent studies.

No events of serum sickness were reported during the extension study. No events suggesting immune complex–mediated end-organ damage, including renal failure, hemolytic anemia, serositis (such as peritonitis, pericarditis, and pleuritis), central nervous system manifestations (such as cerebrovascular accidents or transient ischemic attacks), or myocardial ischemic events related to pegvaliase were observed.

### Immunogenicity

All participants developed a PAL IgG response in the parent studies, and the incidence rate was sustained through the long-term extension study. The majority of participants developed a PAL IgM response, which decreased over time, with incidence rates of 76.6% at Week 24, 50.0% at Week 48, and 46.9% at Week 120. Participants developed a transient anti-PEG response, with the incidence of PEG IgM and PEG IgG peaking at Week 8 and then decreasing to baseline levels by Week 36 of pegvaliase treatment. The incidence rate of NAb increased from baseline, with the majority of participants developing a NAb response that was sustained during long-term treatment; 23.4% of participants were positive at Week 24, 48.2% at Week 48, and 71.4% at Week 120 of treatment.

The frequency of HAEs was highest during early treatment when the incidence rates of PAL IgM, PEG IgM, and PEG IgG antibodies were highest (Fig. 2). The number of HAEs per study week decreased over time, as incidence rates of PEG IgM, PEG IgG, and PAL IgM antibodies decreased, and incidence rates of PAL IgG and NAb antibodies were sustained.

## Discussion

The results from this phase 2 extension study indicate that pegvaliase produced a meaningful and sustained reduction in mean plasma Phe concentration in adults with PKU. The mean decrease in plasma Phe levels to < 600 μmol/L was sustained through 264 weeks (approximately 5 years) of treatment with 256 total patient-years of exposure.

At Week 48 of treatment, almost half (45.9%) of participants had achieved a plasma Phe concentration of ≤360 μmol/L (the recommended upper limit in the United States established by ACMG), and a majority (57.4%) of participants achieved a plasma Phe concentration of ≤600 μmol/L (the recommended upper limit established by the European Society for Phenylketonuria and Allied Disorders for adults) at least once during the extension study [2, 9]. These proportions of participants remained similar throughout most assessment points during long-term pegvaliase treatment.

Total protein intake at baseline (mean 69.4 g/day) reported by study participants was substantially higher than the 6 g/day of natural protein consumed by PKU patients with severe disease. Most study participants were not following a Phe-restricted diet and had protein intake similar to the intake recommended for the unaffected population in the US (about 56 g/day protein for a 70-kg adult) [16, 20]. With pegvaliase treatment, this study population was able to experience sustained plasma Phe reductions with relatively high protein intake at baseline and throughout the study, particularly during Weeks 168–216.

There were no new safety signals detected during the extension study; the AEs that occurred in the extension study were similar to the AEs reported in the parent studies, including those that led to participants discontinuing pegvaliase early (i.e., acute systemic hypersensitivity event, pruritus, and presyncope in one subject each, and arthralgia and peripheral neuropathy in one subject) in the extension study. Event rates for AEs were higher during early treatment in parent studies, when participants were initiating treatment and increasing the dosage of pegvaliase. The frequency of AEs decreased in participants continuing long-term treatment, when the maintenance dose of pegvaliase was achieved. Most participants had AEs limited to mild or moderate severity, all of which resolved.

The safety and immunogenicity results presented here were consistent with previous pegvaliase study reports [13, 14, 21]. As pegvaliase is a bacterial protein, HAEs due to an immune response were expected [20, 22]. The frequency of HAEs was highest during early treatment in the parent studies, when the incidence of PAL IgM, PEG IgM, and PEG IgG antibodies were highest. Overall, the number of HAEs per study week decreased over time as the incidence of PAL IgM, PEG IgM, and PEG IgG antibodies decreased and the incidence of PAL IgG and NAb antibodies increased. The initial antibody response, comprised heavily of pentameric IgM (PEG IgM and PAL IgM), appears to be more efficient at complement activation than the PAL IgG-dominant antibody response that occurs over time [20, 23, 24]. Anti-PEG antibodies are thought to bind to unobscured PEG epitopes on the surface of pegvaliase and activate complement by bringing multiple receptors of the fragment crystallizable (Fc) region of the antibody in close proximity to each other [20, 24]. Together, these observations, in conjunction with the lack of drug-specific IgE positivity, suggest that the likely mechanism of hypersensitivity reactions during therapy with pegvaliase is type III immune complex–mediated hypersensitivity [20, 24].

The magnitude of immune response exhibited by an individual influences the dosage needed to achieve blood Phe reductions, likely due to immune-mediated clearance. A wide range of pegvaliase doses were used for plasma Phe reductions in the extension study, and all participants developed an antibody response to pegvaliase. Subjects with lower antibody responses experienced greater blood Phe reductions in early treatment. As the immune response matures and the pegvaliase dose increases, subjects with a more robust antibody response also experience plasma Phe reductions [10, 16, 25, 26].

The design of this study was open-label, with no placebo or comparator group. Participants were directed to maintain a stable diet monitored by diet diary entries. However, due to the long duration of the study, some variations in diet could have contributed to variability in plasma Phe, and data were not collected to quantify changes in protein intake from medical food versus natural protein. Variability in the Phe levels at later study timepoints likely results from a smaller available sample size due to withdrawals and to participants not yet reaching timepoints as of data cutoff date. A limitation in the descriptive summary for the phase 2 studies is that individual participant data were not evaluated to assess fluctuations or persistency of results. It will be important to assess these in the future, particularly plasma Phe efficacy responses. Individuals with fluctuations in plasma Phe may be difficult to detect, as mean population analyses appear relatively stable over time. In addition, efficacy analyses of achievement of plasma Phe thresholds included participants who reached a specified threshold at least once during the study and did not measure persistency of that achievement.

Many participants continued pegvaliase treatment for an extended period of time. As of October 2016, 66 and 39% of participants who enrolled in the extension study had reached ≥2 years and ≥ 4 years of pegvaliase treatment, respectively. Currently, about half of the participants remain in the extension study, 20 participants discontinued (primarily due to participant or physician decision), and 11 participants completed the study and subsequently enrolled in a phase 3 pegvaliase study. Phase 3 clinical studies have examined the safety and efficacy of pegvaliase in a randomized withdrawal trial [15] and a long-term extension study [16] with plasma Phe, neuropsychiatric, dietary protein intake, and safety endpoints [21].

## Conclusions

Pegvaliase may address an unmet need for many individuals with PKU who have difficulty controlling blood Phe concentration. The PAL-003 phase 2 extension study demonstrated substantial efficacy of pegvaliase in maintaining reduced blood Phe concentration with long-term treatment (up to 5 years). Among participants able to continue with long-term treatment, many reached plasma Phe concentrations within guideline-recommended levels. Overall, the safety profile was managable for the majority of subjects, with AEs limited to mild or moderate severity and AE rates decreasing in participants receiving long-term pegvaliase treatment.

### Additional file

Additional file 1: **Table S1.** Participant demographic and baseline characteristics in extension study. **Table S2.** Pegvaliase exposure. **Table S3.** Blood Phe concentration by study week. (DOCX 33 kb)

## Appendix

### PAL-003 investigators and study sites

D. Adams, Albany Medical College; B.K. Burton, Ann & Robert H. Lurie Children’s Hospital of Chicago; D. Dimmock, Medical College of Wisconsin; K. Goodin, Weisskopf Child Evaluation Center; D. Grange, St. Louis Children’s Hospital; C.O. Harding, Oregon Health & Science University; R. Hillman, University of Missouri; H. Levy, Boston Children’s Hospital; N. Longo, University of Utah; W. Rizzo, University of Nebraska Medical Center; N. Shur, Albany Medical College; J.A. Thomas, Children’s Hospital of Colorado; J. Vockley, Children’s Hospital of Pittsburgh; M.P. Wasserstein, Icahn School of Medicine at Mount Sinai; R. Zori, University of Florida.

## Abbreviations

ACMG: American College of Medical Genetics and Genomics
AE: Adverse event
CTCAE: Common Terminology Criteria for Adverse Events
HAE: Hypersensitivity adverse event
IgE: Immunoglobulin E
IgG: Immunoglobulin G
IgM: Immunoglobulin M
MedDRA: Medical Dictionary for Regulatory Activities
NAb: Neutralizing antibody
NIAID/FAAN: National Institute of Allergy and Infectious Diseases/Food Allergy and Anaphylaxis Network
PEG: Polyethylene glycol
Phe: Phenylalanine
PKU: Phenylketonuria
SAE: Serious adverse event
SD: Standard deviation
SMQ: Standardized MedDRA query
