# Supplementary material for: Long-term safety and efficacy of pegvaliase for the treatment of phenylketonuria in adults: combined phase 2 outcomes through PAL-003 extension study
Source: Orphanet J Rare Dis. 2018 Jul 4;13:108. doi: 10.1186/s13023-018-0858-7 (PMC6031112; doi:10.1186/s13023-018-0858-7)
Supplement: Supplementary file 1 — Table S1. Participant demographic and baseline characteristics in extension study. Table S2. Pegvaliase exposure. Table S3. Blood Phe concentration by study week. (DOCX 33 kb) [file 13023_2018_858_MOESM1_ESM.docx]

| **Table S1** Participant demographic and baseline characteristics in extension study | | |
| --- | --- | --- |
|  |  | **Extension study (n=68)** |
| Age at enrollment |  |  |
| Mean (SD), years |  | 28.3 (8.7) |
| Min, max, years |  | 16, 56 |
| <18, n (%) |  | 3 (4.4%) |
| ≥18, n (%) |  | 65 (95.6%) |
| Sex |  |  |
| Female, n (%) |  | 40 (58.8%) |
| Race |  |  |
| White, n (%) |  | 66 (97.1%) |
|  |  |  |
| Weight |  | (n=68) |
| Mean (SD), kg |  | 83.0 (26.4) |
| Min, max, kg |  | 43.0, 186.0 |
| Height |  | (n=63) |
| Mean (SD), cm |  | 167.7 (9.8) |
| Min, max, cm |  | 150.5, 187.5 |
| Body mass index |  | (n=63) |
| Mean (SD), kg/m^2^ |  | 29.2 (8.4) |
| Min, max, kg/m^2^ |  | 17.7, 60.5 |
| <25, n (%) |  | 21 (30.9%) |
| 25 to <30, n (%) |  | 18 (26.5%) |
| ≥30, n (%) |  | 24 (35.3%) |
| Blood Phe |  |  |
| Mean (SD), µmol/L |  | 1022.4 (530.4) |
| Min, max, µmol/L |  | 1.0, 2196.0 |
| Protein intake* |  | (n=35) |
| Mean (SD), g/day |  | 66.4 (31.0) |
| Min, max, g/day |  | 16.0, 138.3 |
| Phe intake** |  | (n=35) |
| Mean (SD), mg/day |  | 2037.1 (1085.1) |
| Min, max, mg/day |  | 513.0, 5124.0 |
| *max* maximum, *min* minimum, *Phe* phenylalanine *SD* standard deviation  *Protein intake includes medical food and natural protein dietary intakes and was calculated as the daily average intake over 3 days prior to the assessment point.  **Phe intake was calculated as the daily average intake over 3 days prior to the assessment.point. Sample size indicated if n<80. | | |

| **Table S2** Pegvaliase exposure |  |  | |  |
| --- | --- | --- | --- | --- |
|  | **Parent study (n=80)** | | **Extension study (n=68)** | |
| Daily dose, mg |  | |  | |
| Mean (SD) | 5.3 (6.8) | | 26.2 (17.9) | |
| Min, max | 0.13, 30.2 | | 3.7, 107.3 | |
| Mean daily dose, n (%) |  | |  | |
| <20 mg/day | 75 (93.8%) | | 29 (42.6%) | |
| ≥20 to <40 mg/day | 5 (6.3%) | | 25 (36.8%) | |
| ≥40 to <60 mg/day | 0 | | 13 (19.1%) | |
| ≥60 mg/day | 0 | | 1 (1.5%) | |
| Dosing frequency, days/week |  | |  | |
| Mean (SD) | 2.5 (1.5) | | 4.5 (1.6) | |
| Min, max | 1.0, 5.0 | | 1.0, 6.9 | |
| Treatment duration, weeks |  | |  | |
| Mean (SD) | 16.4 (5.4) | | 176.5 (98.8) | |
| Min, max | 0.71, 29.0 | | 4.1, 354.3 | |
| Treatment duration, n (%) |  | |  | |
| <1 year | 80 (100%) | | 11 (16.2%) | |
| ≥1 to <2 years | 0 | | 8 (11.8%) | |
| ≥2 to <3 years | 0 | | 6 (8.8%) | |
| ≥3 to <4 years | 0 | | 18 (26.5%) | |
| ≥4 years | 0 | | 25 (36.8%) | |

Daily dose categories were calculated using total drug amount (mg) divided by entire duration (days) of study to allow comparison of dose between studies.

*max* maximum, *min* minimum, *SD* standard deviation

| **Table S3** Blood Phe concentration by study week | | | | |
| --- | --- | --- | --- | --- |
| Study week | **Phase 2 participants (n=80)** | | | |
|  | Blood Phe (µmol/L) | Change from baseline* | |  |
|  |  | µmol/L | % | |
| Baseline (n=80)  Mean (SD)  Median  Min, max | 1302.4 (351.50)  1266.5  249.0, 2214.0 | NA | NA | |
| Week 24 (n=65)  Mean (SD)  Median  Min, max | 807.0 (551.75)  874.0  1.0, 2005.0 | −497.9 (557.40)  −395.0  −2209.0, 209.0 | −37.6 (38.83)  −26.6  −99.9, 14.9 | |
| Week 48 (n=61)  Mean (SD)  Median  Min, max | 541.6 (515.51)  487.0  1.0, 1697.0 | −796.3 (561.62)  −853.0  −2213.0, 536.0 | −58.9 (39.00)  −69.9  −100.0, 50.3 | |
| Week 72 (n=56)  Mean (SD)  Median  Min, max | 481.3 (446.40)  377.0  1.0, 1648.0 | −866.2 (530.59)  −965.5  −2213.0, 304.0 | −62.8 (35.50)  −71.3  −100.0, 31.0 | |
| Week 96 (n=49)  Mean (SD)  Median  Min, max | 502.4 (491.62)  447.0  1.0, 1769.0 | −867.1 (493.44)  −818.0  −2213.0, 214.0 | −64.2 (33.20)  −67.7  −100.0, 16.7 | |
| Week 120 (n=51)  Mean (SD)  Median  Min, max | 372.0 (514.33)  138.0  1.0, 1868.0 | −1000.2 (581.10)  −1076.0  −2213.0, 545.0 | −72.3 (37.46)  −89.9  −100.0, 46.1 | |
| Week 144 (n=45)  Mean (SD)  Median  Min, max | 476.1 (514.33)  282.0  1.0, 1705.0 | −919.4 (595.58)  −1015.0  −2199.0, 259.0 | −64.6 (38.92)  −79.8  −99.9, 26.8 | |
| Week 168 (n=44)  Mean (SD)  Median  Min, max | 476.4 (558.69)  172.5  1.0, 1741.0 | −904.7 (589.94)  −899.5  −2213.0, 261.0 | −65.7 (38.43)  −84.8  −100.0, 22.4 | |
| Week 192 (n=39)  Mean (SD)  Median  Min, max | 552.7 (535.56)  439.0  1.0, 1752.0 | −827.0 (633.58)  −863.0  −2083.0, 796.0 | −57.5 (43.14)  −63.7  −100.0, 83.3 | |
| Week 216 (n=28)  Mean (SD)  Median  Min, max | 589.1 (557.06)  412.0  1.0, 1709.0 | −804.0 (538.48)  −827.0  −2213.0, 154.0 | −59.4 (36.79)  −66.7  −100.0, 9.9 | |
| Week 240 (n=22)  Mean (SD)  Median  Min, max | 398.8 (486.70)  185.0  1.0, 1655.0 | −1060.5 (535.02)  −1147.5  −2213.0, −181.0 | −72.7 (30.81)  −88.6  −100.0, −9.9 | |
| Week 264 (n=19)  Mean (SD)  Median  Min, max | 791.3 (739.46)  626.0  1.0, 2444.0 | −649.9 (594.87)  −650.0  −1639.0, 406.0 | −48.6 (41.25)  −47.8  −99.9, 24.6 | |

All phase 2 data included. Sample size reflects participants with data available at study timepoint; study is ongoing. *max* maximum, *min* minimum, *NA* not applicable, *Phe* phenylalanine, *SD* standard deviation

*Calculated from baseline (treatment-naïve) in the parent study for participants with available measurements at both time points.
